# Temperature-Dependent Oxygen Effect on NMR *D*-$T_2$ Relaxation-Diffusion Correlation of *n*-Alkanes

**DOI:** 10.1007/s00723-016-0830-4

**Published:** 2016-09-19

**Authors:** Igor Shikhov, Christoph H. Arns

**Affiliations:** School of Petroleum Engineering, The University of New South Wales, Sydney, NSW 2052 Australia

## Abstract

Nuclear magnetic resonance (NMR) diffusion-relaxation correlation experiments (*D*-$T_2$) are widely used for the petrophysical characterisation of rocks saturated with petroleum fluids both in situ and for laboratory analyses. The encoding for both diffusion and relaxation offers increased fluid typing contrast by discriminating fluids based on their self-diffusion coefficients, while relaxation times provide information about the interaction of solid and fluid phases and associated confinement geometry (if NMR responses of pure fluids at particular temperature and pressure are known). Petrophysical interpretation of *D*-$T_2$ correlation maps is typically assisted by the “standard alkane line”—a relaxation-diffusion correlation valid for pure normal alkanes and their mixtures in the absence of restrictions to diffusing molecules and effects of internal gradients. This correlation assumes fluids are free from paramagnetic impurities. In situations where fluid samples cannot be maintained at air-free state the diffusion-relaxation response of fluids shift towards shorter relaxation times due to oxygen paramagnetic relaxation enhancement. Interpretation of such a response using the “standard alkane line” would be erroneous and is further complicated by the temperature-dependence of oxygen solubility for each component of the alkane mixture. We propose a diffusion-relaxation correlation suitable for interpretation of low-field NMR *D*-$T_2$ responses of normal alkanes and their mixtures saturating rocks over a broad temperature range, in equilibrium with atmospheric air. We review and where necessary revise existing viscosity-relaxation correlations. Findings are applied to diffusion-relaxation dependencies taking into account the temperature dependence of oxygen solubility and solvent vapour pressure. The effect is demonstrated on a partially saturated carbonate rock.

## Introduction

Nuclear magnetic resonance (NMR) is commonly used for petrophysical evaluation both in the downhole conditions and for laboratory special core analysis. During the past decade two dimensional relaxation-diffusion *D*-$T_2$ NMR techniques have become widely used in the petroleum industry for providing information about fluid types, their distributions and wetting conditions [1, 2].

Petrophysical evaluation of *D*-$T_2$ correlation maps obtained on rock saturated with multiple fluids (water, oil and gas) involves various asymptotic correlations which aim to assist in interpretation. For instance, the two lines commonly found on *D*-$T_2$ maps are the “water diffusion line” and the “standard alkane line” [1, 3]. The latter represents a relaxation-diffusion correlation valid for pure normal alkanes and their mixtures in the absence of restrictions to diffusing molecules and effects of internal gradients. While this correlation holds for pure, degassed fluids over a broad range of temperatures, there are many situations where measurements in equilibrium with atmospheric air have to be performed.

Air contains a paramagnetic agent—oxygen—which dissolves in fluids rather quickly. The effect of dissolved oxygen on the relaxation time of fluids is long-known, [4]. Oxygen shortens longitudinal and transverse relaxation times of liquids due to NMR paramagnetic relaxation enhancement (NMR-PRE). This effect is routinely utilised in physiological magnetic resonance imaging (MRI) to correlate observations to concentration of oxygen in living tissues. Dissolution of gasses in liquids is important in various branches of engineering, e.g. the rate of gas dissolution is important in aerospace engineering and the oxygen content of fluids is a subject of study in environmental engineering and soil science. In chemical and petroleum engineering the presence of oxygen and the effect of oxygen on relaxation times of hydrocarbons is typically considered as unwanted, requiring thorough purification and degassing for proper experimental conditions. On the other hand, in certain circumstances the sequence of lab experiments designed to characterise saturated rock samples may require temporary exposure of the sample or fluids to atmosphere. Equally, air-saturated fluids may be introduced in the well-bore as part of a technological process. In this case it may be beneficial to have quantitative estimates of oxygen impact on NMR relaxation. The often used NMR capacity to type fluids utilises the connection between viscosity and relaxation time. In particular, Zega et al. [5] demonstrated near constant values of the product of viscosity and relaxation time for deoxygenated alkanes. Straley et al. [6] pointed at the correlation of relaxation time of petroleum fluids to viscosity and temperature. Morriss et al. [7] proposed a viscosity-relaxation correlation for stock tank oils (generally assumed to be in equilibrium with air), which was empirically modified by Vinegar et al. [8] to account for temperature-viscosity dependence. Zhang et al. [9] studying live crude oils, pointed also at significant effect on relaxation time of hydrocarbons due to dissolved air. They suggested that the effect of dissolved oxygen may be described by the correlation of [7] since stock tank oils were subject of their study, while oxygen free alkanes follow a rather different trend on a relaxation-viscosity/temperature normalised plot. Lo et al. [3, 10] developed relaxation-viscosity correlations for gas-hydrocarbon mixtures and live oils. A diffusion-relaxation correlation for pure deaerated alkanes was (for the first time) reported. Zhang et al. [11] demonstrated the effect of oxygen on relaxation of light oils; authors emphasized the efficiency of temperature on removing oxygen from the samples. Chen at al. [12] reported oxygen effect on relaxation time of light oils. They noted the importance of bulk relaxation time of hydrocarbon to additional oxygen related relaxation rate. Furthermore, a temperature-dependent oxygen related enhanced relaxation term is reported as a second-degree polynomial of temperature. Freedman et al. [13] reported diffusion-relaxation correlation of some deoxygenated alkanes and their mixtures, Mutina and Hürlimann [14] systematically studied the effect of oxygen on relaxation time of ten crude oils; a viscosity/long-chain hydrocarbon related dependence on oxygen-related relaxation rate is observed. Winkler et al. [15] studied a variety of petroleum fluids containing significant amount of solution gas in oxygen-free state. They, however, discussed impact of oxygen. The shortening relaxation time of crude oils comparing to alkanes was attributed to dissolved oxygen. However, in later years Mutina and Hürlimann [16] and Benamsili et al. [17] attributed that effect rather to high-molecular components of crudes (specifically asphaltenes). Freed [18] introduced scaling law of diffusion and relaxation for alkanes mixtures at elevated pressures and temperatures (in oxygen free state).

Since normal alkanes and alkane mixtures (e.g., Soltrol) are commonly used in petroleum engineering lab experiments to approximate the oil phase, it would be highly beneficial to obtain the diffusion-relaxation correlation, the “alkane line” of *D*-$T_2$ experiment corrected to the effect of dissolved oxygen. To the best of our knowledge, such a correlation has not been published.

In this work we propose a diffusion-relaxation correlation suitable for the interpretation of low-field NMR *D*-$T_2$ responses of normal alkanes and their mixtures saturating rocks in equilibrium with atmospheric air across a broad temperature interval. We take into account the temperature dependence of oxygen solubility and effect of the latter on observed NMR relaxation time. The change in bulk relaxation response due to the presence or absence of oxygen in fluids may significantly affect relaxation time distribution of vuggy saturated porous systems, e.g. natural carbonate rocks. We demonstrate benefits of the proposed correlation for the analysis of *D*-$T_2$ correlation maps using partially saturated carbonate rocks as an example.

## Correlation of Diffusivity, Viscosity and NMR Relaxation

Physical properties of alkanes are known to be a monotonic function of their carbon number, $\mathrm{C}_n$. Each CH$_2$ group contributes almost linearly to a boiling point and density and to a lesser degree to a melting point, Roberts [19]. From the perspective of the petroleum industry other cross-correlations are of high importance, especially those connecting carbon number, viscosity, self-diffusion coefficient and proton relaxation time. The correlation of fluids’ viscosity to diffusion coefficients is relatively straightforward and can be described either with Stokes-Einstein or Bloembergen’s approach [20], while the connection between NMR relaxation time and diffusion may be less obvious. A number of comprehensive discussions have been published, e.g., see Lo et al. [3], Winkler et al.  [15], Chen et al. [21] and Freed [18]. We tested some of these correlations experimentally and present the results below.

### Correlation of Viscosity and Diffusivity

The most common correlations connecting viscosity, diffusivity and temperature are based on the Stokes-Einstein hydrodynamic model (e.g., Blombergen [20]), where a self diffusion coefficient *D* of a spherical particle is correlated with the shear viscosity ${\eta }$:

1 $$\begin{aligned} D = \frac{k_B }{c \pi r} \frac{T_K}{\eta } \; , \end{aligned}$$

where ${k_B}$ is the Boltzmann constant, ${T_K}$ is the absolute temperature, *r* is the effective hydrodynamic radius of a molecule as a sphere, and *c* is a constant equal to four for slip boundary and six for stick boundary. At a given temperature this correlation predicts an inverse dependence between diffusivity and viscosity. Iwanashi et al. [22] studying alkane homologs ($\mathrm{C}_5$–$\mathrm{C}_{14}$) using $^{13}$C NMR, found that a hypothetical radius of normal alkanes (as in Stokes-Einstein model) strongly correlates with their hydrocarbon-chain length. Using viscosity standards Vinegar [8] demonstrated that the following linear correlation holds (*D* in 10$^{-5}$ cm$^2$/s, $T_K$ - temperature in *K* and $\eta$ - viscosity in cP):

2 $$\begin{aligned} D = \frac{1.29}{\eta }\;\frac{T_K}{298K} \;. \end{aligned}$$

The correlation was obtained using measurements on dead oils at 23 $^{\circ }$C and ambient pressure. Winkler et al.  [15] arrived at a slightly different relationship in the case of crude oil containing a substantial amount of dissolved hydrocarbon gas:

3 $$\begin{aligned} D = \frac{2.55}{\eta }\;\frac{T_K}{298K} \;. \end{aligned}$$

Our experimental observations agree with the fact that dynamic viscosity inversely correlates to self-diffusion coefficient of alkanes. However, in this work we mostly rely on published temperature-viscosity correlations of *n*-alkanes, e.g., van Velzen et al. [23], Dymond et al. [24].

### Correlation of Viscosity and Relaxation Time

Estimation of hydrocarbon viscosity in formation (in situ) is one of the key problems of petroleum reservoir engineering. NMR is the only technology capable to do such estimates. The NMR relaxation time of a pure hydrocarbon correlates with fluid viscosity. The theoretical explanation normally involves molecular-level or rather spin-level theories, e.g., Blombergen [20], Benedek and Purcell [25] theories, where bulk relaxation in fluids is attributed mostly to two terms: intra-molecular dipole-dipole and inter-molecular relaxations. The former term is a function of molecular rotational diffusion (responsible for viscosity) while the latter depends on translational diffusion. Since these two terms are independent, the general correlation between diffusivity, viscosity and relaxivity is hardly possible, unless we consider a sufficiently narrow class of molecular structures, e.g. higher alkanes, narrow range of aromatics, etc. Chiarotti et al. [4] and later Chen et al. [12] and Mutina and Hürlimann [14] attributed a discrepancy between published bulk fluid relaxation times to paramagnetism of dissolved oxygen. Thus, the observed relaxation rate $1/T_{1,2\;\mathrm{obs}}$ is the linear sum of two processes, bulk relaxation time of pure fluid, $T_{1,2B\;\mathrm{pure}}$, defined by dipolar interactions and rotational coupling of spins and relaxation due to paramagnetic species—oxygen gas in this study, ${T_{1,2\;\mathrm{O}_2}}$:

4 $$\begin{aligned} \frac{1}{T_{1,2\;\mathrm{obs}}} = \frac{1}{T_{1,2B\;\mathrm{pure}}} + \frac{1}{T_{1,2\;\mathrm{O}_2}} \; . \end{aligned}$$

For liquid normal alkanes under typical ambient conditions (at atmospheric pressure and temperature interval of interest, from approx. 0 $^{\circ }$C to about 100 $^{\circ }$C) due to the motional narrowing $T_1=$ $T_2$, therefore, here and below a notation for relaxation time $T_{1,2}$ is used and log-mean value where appropriate is assumed. The application of low-field NMR to correlate proton relaxation to crude oil viscosity was pioneered by Brown [26]. The important work in context of this paper has been done by Kashaev et al.  [27] who observed a near constant relationship between relaxation and viscosity for *n*-alkanes, so that $T_1 \propto 1 / \eta$. Straley et al. [6] and Morris et al. [7], based on an analysis of 66 stock crude samples and viscosity standards, established the following correlation between log-mean relaxation time and dynamic viscosity at ambient temperature, often called Morriss correlation (here and below the relaxation time, $T_{1,2}$, is in seconds, viscosity, $\eta$, is in cP and temperature, $T_K$ is in *K* and the appropriate units of prefactors apply):

5 $$\begin{aligned} T_{1,2} = 1.200/ \eta ^{0.9}\; \; . \end{aligned}$$

The very similar form of an equation, which includes a temperature term, was proposed by Vinegar [8]:

6 $$\begin{aligned} T_{1,2} = (1.200/ \eta )\;(T_K/298K)\; \; . \end{aligned}$$

Zhang [9] arrived an expression which generally combines the two correlations above for hydrocarbons saturated with air:

7 $$\begin{aligned} T_{1,2} \; = (1.200/ \eta ^{0.9})\;(T_K/ 298K)^{0.9} \; \; , \end{aligned}$$

and for degassed oxygen-free alkanes:

8 $$\begin{aligned} T_{1,2} \; = (2.125/ \eta ^{1.0})\;(T_K/ 298K)^{1.0} \; \; . \end{aligned}$$

However, there is a discrepancy in literature regarding Eq. 5 in whether it applies to air-saturated hydrocarbons (Zhang et al. [9]) or relates to oxygen-free state (Deng et al. [28]).

**Fig. 1:**
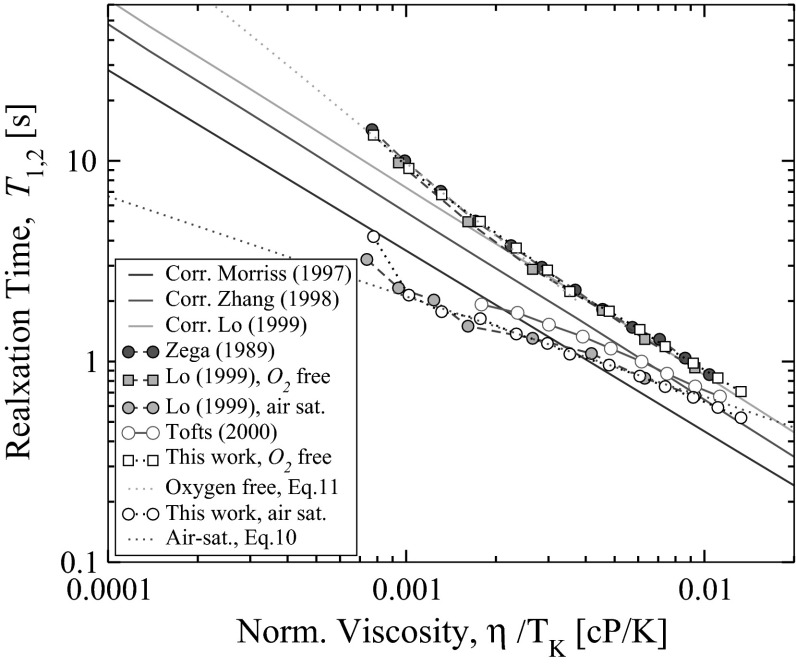
Comparison of experimental relaxation-normalised viscosity data of *n*-alkanes at arbitrary temperature to published sets, both in oxygen-free and air-saturated states (see Tables 1 and 2)

Lo et al. [3] reported a modified correlation for degassed oxygen-free alkanes, which is widely accepted, [12, 15]:

9 $$\begin{aligned} T_{1,2} \; = (2.848 / \eta ^{1.0})\;(T_K/ 298K)^{1.0} \; \; . \end{aligned}$$

Figure 1 demonstrates several published as well as our measured relaxation-time data sets obtained for *n*-alkanes in oxygen-free and air-saturated states. The three solid lines are: modified Morriss correlation [9], Zhang et al. [9] and Lo [10] correlations. It is worth adding that the temperature dependence for the equations above (Eqs. 6–9) was added empirically, while most of the measurements have been performed at a fixed temperature on a variety of fluids to obtain diversity in normalised viscosities $\eta /T_K$. One can see how poorly the correlation of Morriss/Zhang, Eq. 7 approximates air saturated *n*-alkanes. Also note the Tofts et al. [29] data, which are claimed to be obtained on deoxygenated samples, while in reality closely follows air-saturated data sets. Our data shows a very good match with values published by Zega et al. [5], Zhang et al. [9] and Lo [10], both for cases of air saturated and oxygen-free alkanes, see Fig. 1; we added as dashed line an empirical fit to air-saturated alkanes, which holds for the interval of normalised viscosities $\eta /T_K$ of 0.01–0.001:

10 $$\begin{aligned} T_{1,2} \; = (1.15/ \eta ^{0.5})\;(T_K / 298K)^{0.5} \; \; . \end{aligned}$$

Our measurements at ambient conditions and at elevated temperatures performed on pure alkanes, oxygen in which was substituted by introducing pure nitrogen, resulted in a correlation identical to the one reported by Lo et al. [3], restricting comparison to higher alkanes ($\mathrm{C}_{9+}$), Fig. 1. Light alkanes, $\mathrm{C}_5$–$\mathrm{C}_8$, also perfectly match Lo et al. data. However, both sets substantially deviate from the “standard alkane line” correlation, Eq. 9. Fitting oxygen-free $\mathrm{C}_5$–$\mathrm{C}_8$ data requires a higher exponent (shown on the Fig. 1 as a dashed orange line):

11 $$\begin{aligned} T_{1,2} \; = (2.848 / \eta ^{1.3})\;(T_K/ 298K)^{1.3} \; \; . \end{aligned}$$

Regarding air-saturated alkanes, we should point out that though reasonably similar value of relaxation time for air-saturated *n*-pentane is obtained in this work comparing to one reported by Lo [10] (3.8 vs 3.2 s), the temperature conditions are different (22.6 vs 30 $^{\circ }$C). At the latter temperature we obtained twofold higher $T_2$ value of 5.5 s. In our opinion this discrepancy may occur due to different experimental conditions, in particular, sample head pressure. We kept total pressure (fluid vapor and air) at 1 atm., while little excess of head pressure results in substantial change of relaxation time.

**Table 1:** Relaxation time of *n*-alkanes in oxygen-free state

Alkane	This work 2016	Tofts et al. [29]	“Alkane line”	Kashaev et al. [27]	Zega et al. [5]	Lo [10]
Relaxation	$T_2$	$T_1$	$T_2$	$T_2$	$T_2$	$T_1$
Field, $B_o$ (MHz)	2	60	2	...	32	2
Gas state	$\mathrm{O}_2$ free	$\mathrm{O}_2$ free	$\mathrm{O}_2$ free	$\mathrm{O}_2$ free	$\mathrm{O}_2$ free	$\mathrm{O}_2$ free
Temperature ($^{\circ }$C)	22.6	22	22.5	22	25	30
*n*-$\mathrm{C}_{5}\mathrm{H}_{12}$	13.44	–	9.46	14.00	14.30	–
*n*-$\mathrm{C}_{6}\mathrm{H}_{14}$	9.16	–	8.33	8.90	10.00	9.78
*n*-$\mathrm{C}_{7}\mathrm{H}_{16}$	6.78	–	6.22	6.80	7.05	–
*n*-$\mathrm{C}_{8}\mathrm{H}_{18}$	4.99	1.92	4.50	4.90	5.01	4.97
*n*-$\mathrm{C}_{9}\mathrm{H}_{20}$	3.68	1.75	3.40	3.90	3.78	–
*n*-$\mathrm{C}_{10}\mathrm{H}_{22}$	2.86	1.53	2.65	3.00	2.95	2.88
*n*-$\mathrm{C}_{11}\mathrm{H}_{24}$	2.23	1.33	2.12	2.30	2.27	–
*n*-$\mathrm{C}_{12}\mathrm{H}_{26}$	1.78	1.16	1.66	1.90	1.82	1.80
*n*-$\mathrm{C}_{13}\mathrm{H}_{28}$	1.44	1.00	1.35	1.50	1.48	–
*n*-$\mathrm{C}_{14}\mathrm{H}_{30}$	1.19	0.87	1.00	1.20	1.29	1.29
*n*-$\mathrm{C}_{15}\mathrm{H}_{32}$	0.98	0.75	0.87	1.00	1.04	–
*n*-$\mathrm{C}_{16}\mathrm{H}_{34}$	0.83	0.67	0.72	0.80	0.86	0.93
*n*-$\mathrm{C}_{17}\mathrm{H}_{36}$	0.71	–	–	–	–	–

### Correlation of Diffusivity and Relaxation Time

Woessner [30] reported diffusion-relaxation of alkanes, $D/T_1 \propto$ const. Since the *D*-$T_2$ technique was introduced for oil and gas applications diffusion-relaxation relationships of hydrocarbons has become practically important for reservoir fluid typing. That correlation for *n*-alkanes, often called the “standard alkane line” describes the relation between the diffusion coefficient and relaxation time of pure alkanes. Because viscosity and diffusivity of alkanes are connected, as well as NMR relaxation also is a linear function of viscosity, then viscosity can be expressed as following:

12 $$\begin{aligned} \eta _0 = \frac{a\;T}{T_{1,2}} = \frac{b\;T}{D} \; => \; D = \frac{b}{a} \; {T_{1,2}} \; . \end{aligned}$$

Note that the temperature-dependence vanishes in the Eq. 12, thus the diffusion-relaxation time correlation for pure alkanes is valid across a broad range of temperatures. Lo et al. [3] reported the proportionality coefficient *b* as $4.69~\times ~10^{-8}$ ($T_K$/$\eta$) and one slightly different based solely on experimental results: $5.05~\times ~10^{-8}$ ($T_K$/$\eta$). Hirasaki et al. [31] reported the ratio *a*/*b* for alkanes to be *a*/*b* = $0.528~\times ~10^{-9}$ $\mathrm{m}^2/\mathrm{s}^2$ and for crude oils $1.26~\times ~10^{-9}$ $\mathrm{m}^2/\mathrm{s}^2$. The combination of Eqs. 2 and 9 results in $a/b = 0.453~\times ~10^{-9}$, while the value reported by this group, [3, 10], is $0.49~\times ~10^{-9}$. The prefactor *a*/*b* in Eq.12 reported in literature varies slightly. One generally accepted as standard across the majority of publications is the following:

13 $$\begin{aligned} D = (0.5~\times ~10^{-9}\; \mathrm{m}^2/\mathrm{s}^2) \; T_2 \;. \end{aligned}$$

While crude oils are outside the scope of this paper, it is worth adding that such complex fluids may not follow correlation developed for alkanes. For instance, studying crude oils with *D*-$T_2$ NMR Hürlimann [32] observed a more general power law relationship for $T_2$ and *D*. Across the samples the exponent, $\xi$, varies from 0.5 to as high as 4.9.

14 $$\begin{aligned} D \propto T_2\;^\xi . \end{aligned}$$

Korb et al. [33] demonstrated, that the presence of asphaltenes changes diffusion-relaxation relationship in crude oils to $D \propto \sqrt{T_2}$.

## Oxygen Effect on Diffusion-Relaxation Correlation

### Paramagnetic Relaxation Enhancement

Oxygen is known to be a paramagnetic gas. Consequently, the $^1$H NMR relaxation of bulk hydrogen-rich fluids is affected by dissolved oxygen, which shortens the relaxation time of a hosting liquid. The problem of proton relaxation in liquid water containing dissolved oxygen has been considered in a number of works, e.g., Chiarotti et al. [4], Parker and Harmon [34]. The latter found that the modified theory of Torrey [35] describing relaxation by dipolar translational diffusion (i.e scalar coupling effect is negligible) is sufficient to describe the effect of dissolved gaseous oxygen on relaxation in water and potentially in liquids in a more broad sense. Chen et al. [12] and Hürlimann et al. [14] suggested a simple concept of oxygen relaxation enhancement by considering observed relaxation time as the arithmetic sum of two relaxation terms, see Eq. 4: (1) bulk relaxation of pure deoxygenated fluid, 1/$T_{1,2B \; \mathrm{pure} }$ and (2) an oxygen enhanced relaxation term, 1/$T_{1,2 \; \mathrm{O}_2}$. The latter work [14] reported $T_{1,2 \; \mathrm{O}_2}$ between 2.5 and 8.3 s for ten stock tank oils exposed to the atmosphere, with virtually no oxygen effect for components faster than 100 ms. One may attempt to express the oxygen-related relaxation term $T_{1,2\;\mathrm{O}_2}$ as a function of oxygen molecules concentration, $n_{\mathrm{O}_2}$, oxygen diffusion transport in a host fluid, ${D^L_{\mathrm{O}_2}}$ (or transfer coefficient $k_L$) and viscosity of a fluid, $\eta _L$:

15 $$\begin{aligned} \frac{1}{T_{1,2\;\mathrm{O}_2}} = F(n_{\mathrm{O}_2}, D^L_{\mathrm{O}_2}, \eta _L) \; . \end{aligned}$$

Parker and Harmon [34] expressed oxygen-enhanced relaxation rate as a linear function of two terms dependent on concentration of oxygen molecules and a mutual diffusion coefficient:

16 $$\begin{aligned} \frac{1}{T_{1\;w,\mathrm{O}_2}} = \frac{c_1\;n_{\mathrm{O}_2}}{D_{w,\mathrm{O}_2}}\left( c_2\;+\;\frac{c_3}{\sqrt{D_{w,\mathrm{O}_2}}}\right) \; \;, \end{aligned}$$

where $D_{w,\mathrm{O}_2}$ is the mutual diffusion coefficient of oxygen and water, $n_{\mathrm{O}_2}$ the number of oxygen molecules dissolved in water and $\mathrm{C}_1$, $\mathrm{C}_2$, $\mathrm{C}_3$ combines such values like mean square magnetic moment of electron, $<\mu _s^2>$, effective molecular diameter, $\sigma _w$, gyromagnetic ratio of proton, $\gamma _p$, and so on. The anomalously strong impact on NMR relaxation of oxygen dissolved in liquids, even though the concentration of that gas in water at normal conditions typically is about 10–15 ppm, is due to unpaired electronic spin (assuming oxygen is in a triplet ground state, $^3\Sigma,\, S=1$). The strength of paramagnetic relaxation term in Eq. 4, $1/T_{1,2\;\mathrm{O}_2}$ is governed by the product $\gamma ^2\;\gamma _e^2$, where $\gamma _e$ is the electron gyromagnetic ratio, which is 660 times greater than that of proton. This makes this relaxation mechanism significant even at low oxygen concentrations. Thus, the enhanced relaxation rate is defined mainly by unpaired electron spin, also proportional to the concentration of paramagnetic species and may also dependent on viscosity of solvent liquid [36] (using the same abbreviation as in Eq. 16 and rearranging noting Eq. 12):

17 $$\begin{aligned} \frac{1}{T}_{1,2\;\mathrm{O}_2}(T) = \frac{a\;n_{\mathrm{O}_2}(T) \; \gamma _p^2\gamma _e^2 \; \eta _L(T)}{T} = \frac{b\;n_{\mathrm{O}_2}(T)\;}{T_{1,2\;\mathrm{pure}}(T)} \;. \end{aligned}$$

**Fig. 2:**
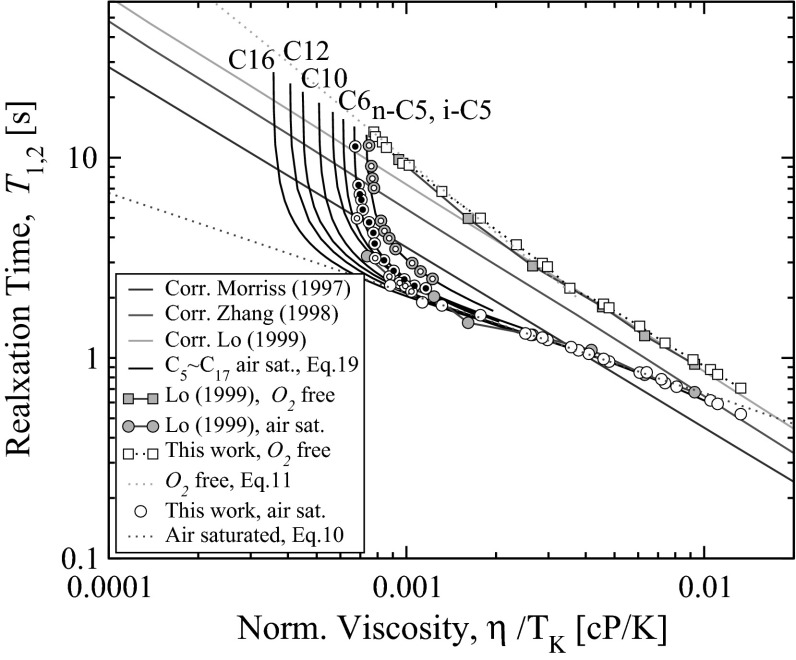
Experimental relaxation-normalised viscosity data of *n*-alkanes (this work, measurements at various temperatures and Lo [10] at ambient). *Open* and *closed double-circled symbols* correspond to our experimental data obtained at various temperatures. The fit with proposed empirical correlation for individual air-saturated alkanes, Eq. 10 shown with annotations for $\mathrm{C}_5$, $\mathrm{C}_6$, $\mathrm{C}_{10}$, $\mathrm{C}_{12}$ and $\mathrm{C}_{16}$, (for $\mathrm{C}_7$ and $\mathrm{C}_8$ the annotations are skipped). In this *plot* iso-pentane data used in addition to normal alkanes $\mathrm{C}_5$–$\mathrm{C}_{17}$

While the theory and findings relative to oxygen paramagnetic enhancement in water are applicable to hydrocarbons, the magnitude of the effect in light hydrocarbons like alkanes is even more pronounced since oxygen solubility is higher (400 ppm at ambient conditions). We observed a 2.5-fold difference in transverse relaxation times between deoxygenated and air-saturated state of *n*-decane, threefold for *n*-octane, fourfold for *n*-hexane and *n*-heptane, see Table 2 and Fig. 2. It is worth mentioning that at $25~^{\circ }$C the “standard alkane line” correlation underestimates relaxation time of light alkanes, e.g. 9.6 s for pentane instead of 14 s.

**Table 2:** Relaxation time of alkanes in equilibrium with air

Alkane	This work 2016	This work 2016	Lo [10]	Lo [10]
Relaxation	$T_2$	$T_2$ ratio of	$T_1$	$T_1$ ratio of
Field, $B_o$	2 MHz	Air sat.: $\mathrm{O}_2$-free	2 MHz	Air sat.: $\mathrm{O}_2$-free
Temperature ($^{\circ }$C)	22.6	22.6	25	25
*n*-$\mathrm{C}_{5}\mathrm{H}_{12}$	4.19	1:3.21	3.23	1:4.47
*n*-$\mathrm{C}_{6}\mathrm{H}_{14}$	2.14	1:4.28	2.33	1:4.21
*n*-$\mathrm{C}_{7}\mathrm{H}_{16}$	1.77	1:3.83	2.03	1:3.48
*n*-$\mathrm{C}_{8}\mathrm{H}_{18}$	1.64	1:3.05	1.50	1:3.31
*n*-$\mathrm{C}_{9}\mathrm{H}_{20}$	1.37	1:2.68	–	–
*n*-$\mathrm{C}_{10}\mathrm{H}_{22}$	1.23	1:2.32	1.31	1:2.21
*n*-$\mathrm{C}_{11}\mathrm{H}_{24}$	1.09	1:2.05	–	–
*n*-$\mathrm{C}_{12}\mathrm{H}_{26}$	0.96	1:1.85	1.10	1:1.64
*n*-$\mathrm{C}_{13}\mathrm{H}_{28}$	0.85	1:1.71	–	–
*n*-$\mathrm{C}_{14}\mathrm{H}_{30}$	0.75	1:1.58	0.83	1:1.56
*n*-$\mathrm{C}_{15}\mathrm{H}_{32}$	0.66	1:1.48	–	–
*n*-$\mathrm{C}_{16}\mathrm{H}_{34}$	0.59	1:1.41	0.68	1:1.38
*n*-$\mathrm{C}_{17}\mathrm{H}_{36}$	0.52	1:1.35	–	–

### Concentration of Oxygen in Alkanes

Equation 17 suggests a linear dependence of PRE on oxygen concentration in liquids. Here we evaluate oxygen concentration at atmospheric pressure as a function of temperature and carbon number. At normal atmospheric conditions air contains about 20.9 % of oxygen by weight. Its maximum equilibrium fraction in water may reach 36 % since it is more soluble than nitrogen. Apparently, the solubility of air in alkanes is a function of carbon number or viscosity. Solubility of gases is normally expressed either as Oswald index, $L_1$, in units of gram of solute gas per litre of fluid solvent or as a molar fraction $x_1$. Oxygen and nitrogen solubility in alkanes is much higher than in water. At 100 % air saturation solubility of oxygen in water at $30\,^{\circ }$C is 7.54 and 8.56 mg/L at $23\,^{\circ }$C. 1 l of water may hold about 16 mg of air, while alkanes in $\mathrm{C}_6$–$\mathrm{C}_{16}$ interval can retain 10–20 times more. The reported [37] relative solubility strength (relating pure oil to pure water) is 11.1, 7.7 and 6.5 for *n*-heptane, *n*-dodecane and *n*-hexadecane respectively.

At atmospheric pressure, temperature-dependent fractional solubility of oxygen is limited by solvent vapour pressure and reaches zero at a boiling point. Thus, the oxygen fraction, $n_{\mathrm{O}_2}$ can be expressed as a product of oxygen solubility $x_1$, oxygen fraction in the air $f_{\mathrm{O}_2}$ and further reduced by vapour air pressure (normalised), $(1 - P_v)/P_\mathrm{atm}$. Using one of the many available expressions for alkanes solubility and solvent partial vapour pressure (e.g. based on Clausius-Clapeyron equation of state in the form of Antoine equation), molar amount of oxygen in the solution, $n_{\mathrm{O}_2}$, can be expressed as following:

18 $$\begin{aligned} n_{\mathrm{O}_2} = f_{\mathrm{O}_2} n\; e^{A_1(T_K) - B_1 Cn} \; (P_\mathrm{atm} - e^{A_2 - B_2 T_{b}/T_K}) / P_\mathrm{atm} \;, \end{aligned}$$

where the first term is oxygen fraction in the air, the second approximates molar solubility $x_1$ following Buttino et al. (1984) and the last term approximates solvent vapour pressure, Smialek [38].

### Viscosity-Relaxation Correlation for Air-Saturated Alkanes

The Arrhenius-style expressions are often used to correlate kinetics of chemical processes as well as can be applied to temperature-dependent physical properties, like viscosity of liquids in the form of Andrade equation. Van Veltzen et al. (1972) poroposed a modification to improve the accuracy of viscosity predictions using a specific known arbitrary point $T_0$ at which viscosity is equal to 1 cP; viscosity-temperature correlation:

19 $$\begin{aligned} \eta (T_K) = e^{B\;(1/T_K\;-\;1/T_0)} \; \;. \end{aligned}$$

We propose an empirical correlation for the oxygen related relaxation term $T_{1,2\;\mathrm{O}_2}$, based on the fact that for each *n*-alkane within the range of carbon numbers $\mathrm{C}_n$ from 3 to 19 that term is zero at a boiling temperature $T_{b,Cn}$. Assuming that relaxation enhancement caused by oxygen linearly decreases over temperature, normalised by the temperature interval over which the particular alkane is liquid, we arrived at equation Eq. 20. It has some similarity to one proposed by van Velzen et al. [23], but utilises melting temperature as a reference point:

20 $$\begin{aligned} \frac{1}{T_{1,2\;\mathrm{O}_2}(T_K,C_n)} = \alpha \; \left( \frac{T_{b,\;Cn} - T_K}{T_{b,\;Cn} - T_{m,\;Cn}}\right) ^\beta \; \;. \end{aligned}$$

Combining Eq. 20 with Eq. 4 we can obtain an empirical correlation for the observed relaxation time of air-saturated alkanes as function of temperature and carbon number.

21 $$\begin{aligned} \frac{1}{T_{1,2\;\mathrm{obs}}(T_K,C_n)} = \frac{1}{T_{1,2\;B}(T_K,C_n)} \;+\; \alpha \; \left( \frac{T_{b,\;Cn} - T}{T_{b,\;Cn} - T_{m,\;Cn}}\right) ^\beta \; \;. \end{aligned}$$

Here $T_b$ is a boiling temperature and $T_m$ is a melting temperature of a specific *n*-alkane (depending on $\mathrm{C}_n$) and $\alpha =$ 0.95, $\beta =$ 0.5. To predict relaxation time in air-saturated state Eq. 20 assumes that for pure deoxygenated *n*-alkanes the linear correlation between relaxation time and viscosity holds over the whole temperature interval from the melting to the boiling point and the relaxation time $T_{1,2}(T,\;C_n)$ and viscosity $\eta (T,\;C_n)$ in that state are known. Figure 2 shows viscosity-relaxation values of air-saturated alkanes. Note a region approximately between 0.001 and 0.01 values of $\eta$/$T_K$ where a linear relationship with relaxation holds. At lower values of $\eta$/$T_K$ (closer to boiling temperatures) the relationship becomes increasingly specific to the alkane carbon number. The correlation Eq. 21 predicts rather a non-trivial shape of iso-thermal curves for air-saturates alkanes.

### Diffusion-Relaxation Correlation for Air-Saturated Alkanes

Since relaxation time and self diffusion coefficient are both proportional to viscosity-to-temperature ratio ($T_{1,2} \propto \eta /T_k$ and $D_{0} \propto \eta /T_k$), the diffusion-relaxation correlation Eq. 13 “standard alkane line” holds for different temperatures.

**Fig. 3:**
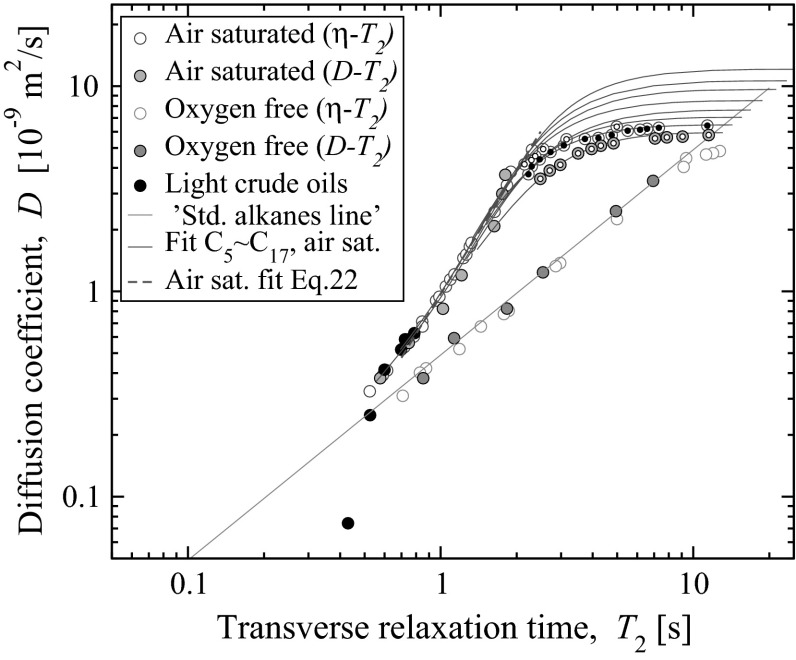
Experimental diffusion and $T_2$ relaxation data of air-saturated and oxygen-free *n*-alkanes at various temperatures obtained from CPMG experiments and viscosity to diffusion conversion (same as Fig.2) and direct *D*-$T_2$ measurements (log-mean values of maps depicted at Fig.4). *Open* and *closed* *double-circled symbols* correspond to our experimental data obtained at various temperatures. Here an iso-pentane data set is added to *n*-$\mathrm{C}_5$–*n*-$\mathrm{C}_{17}$

However, the diffusion-relaxation correlation of oxygen-saturated alkanes has apparent temperature dependence, subject to a particular carbon number, $\mathrm{C}_n$. One correlation which is independent of alkane type or $\mathrm{C}_n$, can be obtained by transforming the viscosity-relaxation correlation observed for air-saturated alkanes over the normalised viscosity interval 0.001–0.01 *cP* / *K*, Eq. 10. Substituting $\eta$ from Eq. 2 the result is

22 $$\begin{aligned} D = 0.975 \cdot T_{1,2\;\mathrm{obs}}^2 \; . \end{aligned}$$

This correlation is independent of temperature and carbon number over the interval of diffusivities 0.6–6 $\times$ 10$^{-9}$ m$^2$/s. Figure 3 demonstrates that this correlation is valid in a practically important interval of diffusion values.

## Fluid Samples and Experimental Techniques

### Fluid Samples

Quantitative fluid typing is of particular importance for NMR petrophysics. The common approach to approximate crude oil properties in flooding experiments is to use pure alkanes, their mixture or standard refined oils, like refined kerosene fraction, Soltrol, produced by Chevron Phillips Chem. Co. Here we use normal alkanes, *n*-$\mathrm{C}_5$–*n*-$\mathrm{C}_{17}$, which are liquid in the temperature interval of interest, to study the temperature-dependent paramagnetic relaxation enhancement due to dissolved oxygen. The purity of fluids is at least 98 % and in most cases 99+ %, while the remaining fraction is mostly water. Volume of fluid samples was about 25 cc confined in HDPE bottles. Oxygen-free state was achieved by bubbling the samples by gaseous nitrogen in situ as well as during the CPMG relaxation measurements. The standard approaches to treat samples sensitive to oxygen include removal of saturated air by vacuuming, freeze-pump-thaw and heating. The latter is proposed by Zhang et al. [11], who also demonstrated the significance of oxygen in shortening transverse relaxation times of light crudes. We removed oxygen from fluids by supplying nitrogen into samples during NMR experiments. We found that the nitrogenated sample NMR response is similar to that of a vacuumed sample, but preserves oxygen-free conditions for longer.

### Techniques

While our focus here is the effects of paramagnetic enhancement on *D*-$T_2$ responses, in the course of relaxation data acquisition the ordinary CPMG technique was widely used. These two techniques are long-known and detailed description and theory can be found elsewhere. We used a pulsed field gradient (PFG) *D*-$T_2$ technique implemented with a PGSTE variant for the diffusion encoding [39]. Here we state the acquisition and experimental parameters of these experiments. All measurements were performed with a reasonably short echo-spacing $\tau _E=$200 μs and a mixing time $\Delta =$ 40 ms. The pulsed gradient was applied in 30 steps varying from $G_{\mathrm{ext}}=$ 0.15 G/cm for fast diffusing pentane to $G_{\mathrm{ext}}=$ 65 G/cm for slow diffusing hexadecane. With four scans the signal-to-noise ratio (SNR) was above 400. The NMR measurements were made on a Magritek 2 MHz NMR Rock Core Analyzer using home-built temperature cell in a temperature interval from −15 to 65$^{\circ }$C.

## Experimental Results

### Effect of Dissolved Oxygen on NMR Responses of Bulk Alkanes

**Fig. 4:**
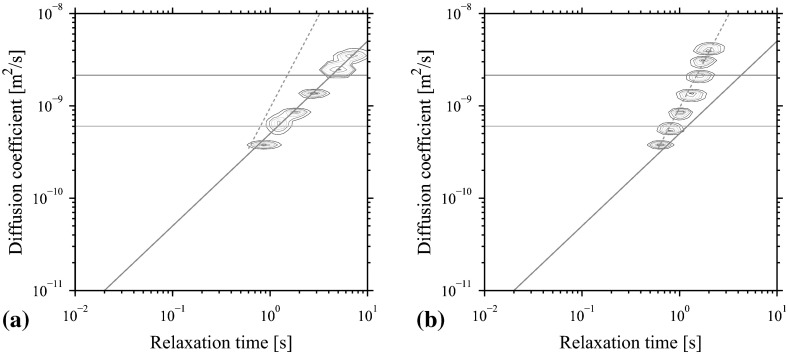
*D*-$T_2$ multi-plots of individually measured *n*-alkanes at arbitrary temperature 22.6$\,^{\circ }\mathrm{C}$: **a** $\mathrm{C}_7$, $\mathrm{C}_8$, $\mathrm{C}_{10}$, $\mathrm{C}_{12}$, $\mathrm{C}_{14}$, $\mathrm{C}_{16}$ in oxygen-free state, **b** $\mathrm{C}_6$, $\mathrm{C}_7$, $\mathrm{C}_8$, $\mathrm{C}_{10}$, $\mathrm{C}_{12}$, $\mathrm{C}_{14}$, $\mathrm{C}_{16}$ in equilibrium state with air. The *solid red line* is the diffusion-relaxation time correlation for alkanes in oxygen-free state. The *dashed red line* follows the proposed correlation for alkanes in oxygen-free state

*D*-$T_2$ experiments were performed on bulk *n*-alkane samples in air-saturated and oxygen-free (nitrogen saturated) states. Initially samples were at equilibrium air saturation. Figure 4a depicts the resulting individual relaxation-diffusion maps, the six components are measured *individually in separate experiments*, on a single multi-plot.

Similarly, Fig. 4b shows a multi-plot of experimental *D*-$T_2$ maps obtained individually on deoxygenated alkane samples, plotted together to demonstrate a trend. *D*-$T_2$ of oxygen-free alkanes follows the expected linear trend of Lo et al. [3]. However, the air-saturated maps significantly shift compared to the “standard alkane line”. The *D*-$T_2$ maps have four indicator lines to assist the interpretation. The horizontal blue line indicates a self-diffusion coefficient of water, here $D_w=$ $2.15~\times ~10^{-9}$ $\mathrm{m}^2/$s and the green line shows a self-diffusion coefficient of Soltrol 130, $D_o=$ $0.60~\times ~10^{-9}$ $\mathrm{m}^2/$s. The inclined red line indicates the “standard alkanes line”, $D_a=$ $0.50~\times ~10^{-9}$ $[\mathrm{m}^2/\mathrm{s}^2]$ $T_{2B}$, where $T_{2B}$ is the relaxation time of a pure alkane. The dashed red line shows the actual scaling power-law fit for air-saturated alkane samples. One can see that the individual responses of air-saturated bulk pure alkanes follow the proposed correlation.

### Effect of Dissolved Oxygen on NMR Responses of Saturated Rocks

In this section we illustrate the effect caused by dissolved oxygen on NMR relaxation in the case of a saturated rock sample. We use a 50.8 mm long (2 in.) Mount Gambier carbonate sample of diameter 25.4 mm (1 in.), which exhibit high porosity, $\phi =$ 52 $\%$. Initially the sample was saturated with 3 $\%$ NaCl brine, then drained with dodecane until $S_w=69\,\%.$ In the second stage the sample was exposed to oxygen free dodecane for several hours. After each step CPMG and PGSTE-CPMG measurements were performed.

**Fig. 5:**
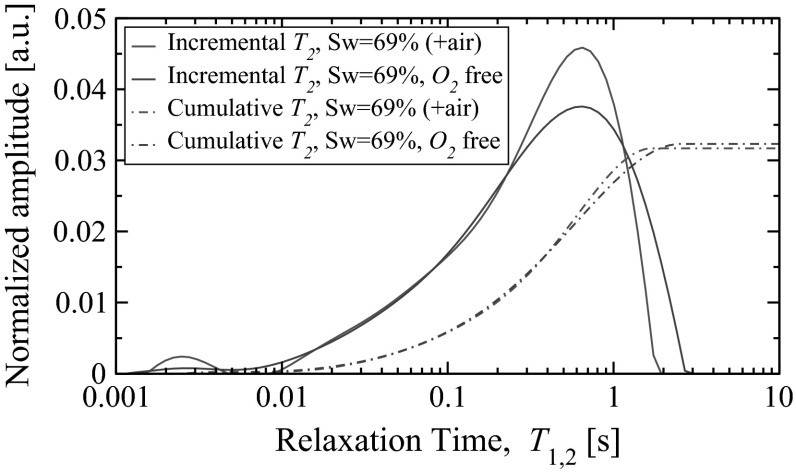
Incremental and normalised cumulative $T_2$ distributions of Mount Gambier carbonate partially saturated with NaCl brine ($S_w=$ 69 $\%$) and dodecane ($S_o=$ 31 $\%$). Results reported for two cases: (1) dodecane is in air saturated state (+air) and (2) dodecane is in oxygen free state ($\mathrm{O}_2$-free)

As expected even at moderate oil saturation, $S_o=31\,\%,$ the observed shift of the $T_2$ response was substantial, see Fig. 5. For instance, the log-mean of the whole $T_2$ distribution increased from 285 to 315 ms. Excluding the part of distribution associated with immobile fluid ($T_2 <$ 92 ms), the shift is even more pronounced: 446 ms in the case of air saturated fluids and 489 ms after exposure to oxygen-free dodecane. The shift due to oxygen is expected to be even stronger if one or both saturating fluids would be deoxygenated before flooding. In the demonstrated case only the connected dodecane phase saturating sample had its oxygen replaced. In particular, at lower oil saturation, $S_o=11\,\%,$ the change in relaxation time was barely observed since practically the whole oil phase is disconnected from the sample’s outer boundary.

**Fig. 6:**
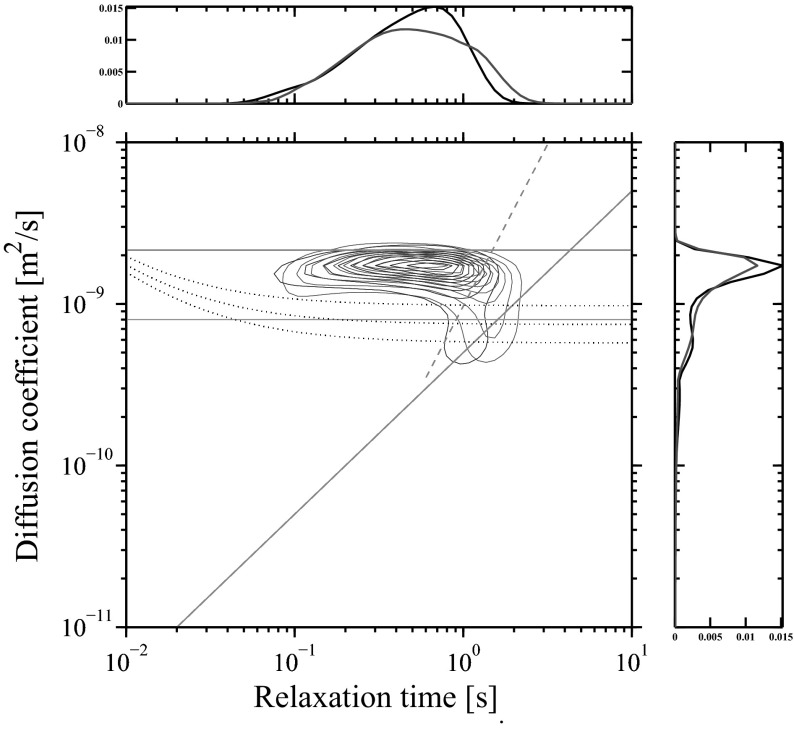
Diffusion-relaxation map showing two overlapped *D*-$T_2$ distributions of Mount Gambier carbonate partially saturated with dodecane in air saturated (blue contours) and oxygen free states (red contours). Interpretation of the map is assisted with three detection limit curves (*dotted black*), the *upper* of which is 10 % limit, *middle* −5 % and the *lower* is 2 % (see details in the text). The *diagonal and horizontal reference lines* are as in Fig. 4

Figure 6 shows a diffusion-relaxation correlation map obtained at the same conditions as described above for CPMG experiments. The map shows the “standard alkane line” $\propto T_2$ (oxygen-free conditions) as well as proposed correlation $\propto T_2^2$ (for air-saturated state). The three nearly parallel dotted black lines show *D* and $T_2$ detection limits of 10, 5 and 2 $\%$ (for the surviving signal attenuation) for the given set of experimental parameters (*G* and $\Delta$), following conceptually Flaum et al. [2] adopted for the PFG variant of *D*-$T_2$ experiment:

23 $$\begin{aligned} D(T_2) = \frac{{\log }(0.02;0.05;0.10)\;-\;\Delta /T_2\;-\;2\tau /T_2}{-G^2\gamma ^2\delta ^2(\Delta - \delta /3)}, \end{aligned}$$

where *G* = 0.60 G/cm, $\Delta =40$ ms, $\delta =$3 ms, $\tau =$100 $\mu$s and $T_1 \simeq T_2$ is assumed. The map demonstrates the apparent shift towards longer relaxation times. In addition, observed responses from air-saturated and oxygen-free dodecane are likely overlapping due to the limited resolution of the inverse Laplace transform, [40, 41]. In addition, Fig. 6 shows projections on diffusion and relaxation domain a technique commonly used within the industry, [42, 43]. Projections demonstrate that the presence of oxygen in fluid(s) affects mainly the relaxation domain, while very little change can be seen in the diffusion projection.

## Discussion and Conclusions

We experimentally observed deviations of both viscosity-relaxation and diffusion-relaxation times of light-end *n*-alkanes ($\mathrm{C}_5$–$\mathrm{C}_8$) or more strictly, lower-end of normalised viscosity, in oxygen-free state from published correlations. The degree of this deviation can be regarded as significant if natural medium and high viscosity oils are not considered. We propose a correction to equation of Lo et al. [3]. However, we belive that the reported deviation may be relevant to all alkanes once their temperature is close enough to corresponding boiling point and may not be described with the typical simplistic correlation. Testing this, however, is outside our technical capacity.

For the case of alkanes in equilibrium with air the existing model provide satisfactory estimates only for the light and medium oils, while the standard set of alkanes ($\mathrm{C}_5$–$\mathrm{C}_{17}$) is poorly correlated. Furthermore, we demonstrated that for that set of alkanes a general correlation without accounting for carbon number is not possible since their boiling temperatures are different.

It is practically useful to evaluate the rate of oxygen back re-saturation process of a fluid sample at a typical lab conditions. While oxygen saturation of a closed sample having air head (ullage) depends on many factors like thermal convection, physical shaking of a sample, pressure and humidity fluctuation in the lab and sample geometry, the observed rate of relaxation time change can be well fitted by the following expression:

24 $$\begin{aligned} T_{2B\;\mathrm{obs}}(t) = T_{2B\;\mathrm{air}\;\mathrm{sat}} + T_{2\;\Delta \;(\mathrm{O}_2)} \; e^{-\alpha t} \;, \end{aligned}$$

where the observed relaxation time of a sample partially equilibrated with air oxygen, $T_{2B\;\mathrm{obs}}(t)$ is the linear sum of two terms: (1) bulk relaxation time of fluid in equilibrium with oxygen and (2) exponential term containing PRE-related additional relaxation time $T_{2\;\Delta \;(\mathrm{O}_2)}$ and $\alpha$—a term describing saturation rate. In particular for water which exhibits at 22.6$~^{\circ }\mathrm{C}$ in oxygen-free state bulk relaxation time of 3.28 s, it drops slowly to 2.89 s in 6 h. It takes full 2  days to fully equilibrate a sample with air, resulting in a relaxation time of 2.48 s. Similarly, *n*-decane experiences a rather fast drop of bulk relaxation time from 2.86 s in oxygen-free state to 2.31 s in 1 h and 1.43 s in 6 h. It takes a very long time to reach the fully air-saturated state precisely without shaking the sample.

The main result of this study is the development of an analytical model providing a temperature dependent diffusion-relaxation correlation in the presence of oxygen contained in the air. The model is supported by experimental measurements using normal alkanes over a temperature range from −15 to 60$~^{\circ }\mathrm{C}$. We demonstrated the effect of oxygen paramagnetic enhancement on relaxation response of saturated carbonate rocks and the usefulness of the proposed correlation for improved interpretation of *D*-$T_2$ correlation maps.
